# Medical Miscellany

**Published:** 1850-01

**Authors:** 


### ARTICLE IX.

### MEDICAL MISCELLANY.

Prof. Shotwell has been transferred to the chair of Materia Medica, made vacant in the Medical College of Ohio, by the death of Pro. Harrison.

Seven female students are in attendance on the lectures in the Eclectic establishment at Syracuse, N. Y.

Dr. E. W. H. Ellis, of Goshen, has been elected Auditor of State, in Indiana.

The Coroner's jury of Boston, after an elaborate investigation of the cause of death, in the case of Dr. Geo. Parkman, returned a verdict accusing Prof. J. W. Webster, of the Boston Medical School, with the murder, who is committed to prison to await his trial.

Dr. Hubbard has been elected Governor of the State of Maine.

Dr. N. D. Benedict has been appointed Superintendent of the N. Y. State Lunatic Asylum at Utica, in place of Dr. Brigham, deceased.

Prof. Paul F. Eve, who has for five years edited with great ability, the Southern Med. and Surg. Journal, has retired from that post, and is succeeded by Dr. I. P. Garvin, formerly associate editor of the same work.— This move, we are pained to learn, has been caused by severe domestic afflictions. Our warmest sympathies are tendered him.

